# Barrier defect in atopic dermatitis – possibilities and limits of basic skin therapy 

**DOI:** 10.5414/ALX02268E

**Published:** 2021-08-27

**Authors:** Lennart M. Roesner, Annice Heratizadeh

**Affiliations:** Department of Immunodermatology and Experimental Allergology, Clinic for Dermatology, Allergology and Venereology, Hannover Medical School, Hannover, Germany

**Keywords:** skin, atopic dermatitis, skin barrier, basic therapy, emollients, primary prevention, secondary prevention, ingredients

## Abstract

The increased permeability of the skin barrier towards environmental factors such as allergens is considered a key factor in the pathogenesis of atopic dermatitis (AD). Strengthening the skin barrier through basic skin therapy represents the basis of any therapy for AD. It is well known that genetic factors as well as the skin inflammation itself contribute to the weakening of the barrier; here, recent studies have led to a deeper understanding of the complex structures of the epidermis. The possibility of counteracting the disease preventively by the use of basic skin therapy from birth on has been studied intensively in recent years. This article summarizes recent findings on the effects of basic skin therapy as a primary and secondary preventive measure.

## Introduction 

As our outer barrier, the skin is continuously exposed to environmental influences and pathogens. Disruption of skin barrier function is known to be one of the major risk factors in the development of allergic diseases such as atopic dermatitis (AD) [1]. The “outside-to-inside” hypothesis describes that permeability of the skin to, for example, irritants, allergens, or microorganisms leads to an exaggerated immune response and sensitization to allergens. Persistent skin inflammation leads to further weakening of barrier function through direct action of various cytokines of the type 2 inflammatory cascade [2], which has become known as the “outside-to-inside-and-back-to-outside” hypothesis. This interplay of barrier, inflammation, and disease development suggests that maintaining the integrity of the skin barrier is critical as a therapeutic goal as well as in primary prevention measures for atopic disease. 

The foundation of therapy in AD consists of a consistent basic skin therapy with active ingredient-free topical preparations which are hydrating and lipid-replenishing at the same time. In this regard, both the German and the European guideline for treatment of AD consented to the strongest and second strongest formulation of a positive recommendation, respectively [3, 4]. 

The basic skin therapy of AD aims at counteracting dehydration of the skin due to an increased transepidermal water loss and a concomitant intensification of the already existing, genetically and/or inflammatory skin barrier disorder [5, 6, 7]. 

## Effects of a basic skin therapy as a primary prevention measure 

Several years ago, first small proof-of-concept studies aimed at a primary preventive effect of a basic skin therapy of several months in allergy-prone infants from birth or from neonatal age until 6 months or 32 weeks of age. In two studies from the USA and Japan, a reduction in the development of AD within the observation period of ~ 30% and 50%, respectively, was observed compared to the non-treated control group [8, 9]. 

These results were a starting point for subsequent larger randomized controlled trials (RCT): in the BEEP (Barrier Enhancement for Eczema Prevention) study, 1,394 infants at high risk of eczema (i.e., eczema in at least 1 first-degree relative) were treated at least once daily or not at all with basic skin therapy in a 1 : 1 randomization during their first year of life [10]. The primary endpoint was the eczema rate at 2 years of age, which was not significantly different between the two study groups (23% versus 25%) after this observation period. Subgroup analyses of filaggrin (FLG) genotype, among other factors, also revealed no seminal differences. 

In another study with a partially similar study design (PreventADALL), 2,397 infants were randomized into four study groups [11]: (1) no intervention, (2) basic skin therapy including bath supplement, (3) early supplemental administration of peanut, cow’s milk, wheat, egg, (4) interventions as in both (2) and (3). Also in this study, no preventive effect was observed at 12 months of age with regard to the development of AD. In 2019, a protocol for a multicenter phase III study from Australia was also published [12]: from birth to 6 months of age, infants with a positive family history regarding allergies are to receive ceramide-containing baseline therapy twice daily (NCT03667651). The primary endpoint refers to the eczema rate at 12 months of age. To date, however, no further results on this have been published in the international clinical trials database *ClinicalTrials.gov*. 

A systematic Cochrane review published in 2021 on the question of primary prevention identified 33 RCTs including 25,827 study patients [13] and concluded, among other things, that a targeted basic skin therapy compared with no basic skin therapy or usual skin care neither influences the risk with regard to the development of eczema at the age of 1 – 2 years nor the time course until the manifestation of eczema. In contrast, the authors consider a higher rate of skin infections as probable under basic skin therapy, and an increased incidence of adverse effects such as an allergic reaction to emollients agent is assumed. 

Consequently, overall, the current evidence base does not yield a primary preventive effect of basic skin therapy in allergy-prone infants on the risk for AD. 

The lack of success of these studies may also be due to the complexity of skin barrier formation. Basic research has added crucial knowledge in recent years that could be incorporated into future approaches to strengthening the barrier. 

The skin barrier is often subdivided into the physical, chemical, immunological, and microbial barriers in analogy to the mucosa of the gut. The physical and chemical barriers are primarily located in the stratum corneum and further in the upper epidermis. In addition to the cell-cell connections of the epidermis (for example, the tight junctions), keratinization is the decisive factor here, in which keratinocytes form various components (keratohyalin granules and lamellar bodies) during progressive differentiation and ultimately form the structure of the stratum corneum as flattened, nucleus-free corneocytes and the cornified envelope. Recent studies suggest that the multiprotein complexes of the mechanistic target of rapamycin (mTOR) play important roles in controlling these processes. Here, mTORC1 is involved in keratinocyte proliferation and initiation of the differentiation program, while the mTORC2 signaling pathway controls terminal differentiation [14]. 

At the structural level, barrier proteins such as filaggrin, loricrin, keratin and corneodesmosin, as well as lipids play crucial roles. Most knowledge to date has been gained on the function of filaggrin. Keratinocytes of the stratum granulosum initially express a protein consisting of 10 – 12 linked filaggrin monomers (profilaggrin), which is incorporated into keratohyalin granules and later proteolytically cleaved into monomers. The monomers bind keratin filaments and finally form the scaffold of corneocytes. In a recent work, the protein zinc α-2-glycoprotein (ZAG) produced by adipocytes and keratinocytes of the skin was identified as a positive regulator of filaggrin expression [15]. ZAG, which is also proclaimed as a biomarker for AD, was also able to positively affect the levels at the epidermal cytokine thymic stromal lymphopoietin (TSLP) in this study. 

The crucial role of filaggrin is underlined by the fact that mutations in the encoding *FLG* gene are considered to be the most common genetic predisposition for AD and that disturbances of the skin barrier and especially of filaggrin production lead to skin inflammation and allergic sensitization. Research approaches to develop innovative therapy therefore also aim at upregulating filaggrin expression [16]. 

As previously mentioned, allergic inflammation of the skin leads to a reduction in barrier integrity, in part by decreasing the expression of barrier proteins. In line with this, it has been currently shown that therapy with dupilumab, which specifically suppresses allergic inflammation by blocking the interleukin (IL)-4 and -13 signaling pathways, also leads to a strengthening of the barrier [17]. In this study, filaggrin and lymphoepithelial Kazal type-related inhibitor (LEKTI) proteins were shown to be significantly increased after only 6 – 8 weeks, and at expression levels equivalent to healthy skin. Epidermally expressed pro-inflammatory cytokines were also measured to be decreased, namely TSLP, IL-15, and IL-25, which in turn may have a positive effect on the skin barrier. 

The tight junctions of the epidermis are a complex of adhesive and structuring proteins that seal cellular interstitial spaces beneath the stratum corneum, thereby controlling the permeability of the skin to water, ions, and solutes. A recent study of primary human keratinocytes and skin explants showed that the cytokine IL-17, which plays a role primarily in the immune response to pathogens, enhances the formation and function of tight junctions in a signal transducer and activator of transcription(STAT)3 pathway-dependent manner. Specifically, the enhancement of barrier function was measured by transepidermal electrical resistance (TEER), and tight junction formation was demonstrated by expression of the protein claudin-4. IL-4, as a key cytokine of allergic inflammation, again blocked these effects [18]. 

## Effects of basic skin therapy as a secondary prevention measure 

In the same year as the above-mentioned proof-of-concept studies for primary prevention, results of a controlled intervention study were published from Norway, which, in the sense of a very early secondary prevention measure, was oriented towards the skin condition of infants aged 6 weeks and investigated the effects of a daily oil bath and a fat emollient for the face in comparison with “normal skin care” on the reduction of xerosis and manifestation of AD [19]. Using a non-validated, semi-quantitative skin score (0 – 4), in a study population of 56 infants in total, “normal” skin was observed significantly more often in the group with intensified basic skin therapy compared to the control group at the age of 6 months, and the infants were less often assigned a skin score (4) of “probably eczema” (4 vs. 19%). Overall, however, the study data with regard to a secondary preventive effect of a basic skin therapy in AD is so extensive that corresponding positive recommendations have been consented in the therapy guidelines for AD and the basic skin therapy is a fixed component of the four-step therapy regimen [3, 4, 20]. In this context, oil baths are to be distinguished from a lipid-replenishing and hydrating basic therapy by means of emollients. In an open RCT (BATHE) including 482 children (mean age 5 years), no additional benefit could be observed by a combined use of regular oil baths with a regular eczema therapy (including regular creaming with an active agent-free topical preparation) based on the subjective severity score of AD (patient-oriented eczema measure (POEM)) [21]. 

A translational study we conducted in collaboration with the Fraunhofer Institute for Experimental Medicine and Toxicology (ITEM) in Hannover, Germany goes beyond the concept of “general secondary prevention” (data unpublished): To test whether basic skin therapy can protect against an episode of AD triggered by an inhalant allergen via the surrounding atmosphere, at least moderately grass pollen-rich IgE-sensitized patients with moderate-to-severe AD were exposed to a grass pollen-rich ambient atmosphere (corresponding to a summer meadow). In a previous study, this had triggered an episode of AD in sensitized patients [22]. While the patients of the intervention group had received a consequent basic skin therapy already 7 days before exposure, the patients of the control group were exposed to the allergen-containing atmosphere without any basic skin therapy. In the control group, a worsening of AD could be observed, while in skin samples from patients with the basic skin therapy, a comparatively significantly reduced number of IL-13 and chemoattractant receptor-homologous molecule expressed on Th2 (CRTh2)-expressing cells could be detected as a sign of a lower Th2 immune response in the skin. Skin microbiome analyses of emollient-treated patients also indicated stabilization of the skin microbiome during grass pollen exposure. 

In summary, according to the current state of studies and corresponding therapy guidelines, a consistent basic skin therapy is recommended for AD. The extent to which this may also have a protective effect against specific environmental influences such as inhalation allergens remains to be investigated in further prospective RCTs. 

## Practical aspects of ingredients of a basic skin therapy in AD 

In the guidelines for AD, barrier-stabilizing ingredients such as the (age-adjusted) addition of urea as well as glycerol are mentioned for a basic skin therapy [4, 20]. Recent innovative approaches with the addition of, for example, bacterial lysates, protein-free plant extracts, flavonoids, saponins, and riboflavins, which are not to be classified as medicinal additives according to the German Drug Law, have also led to the introduction of the additional category “emollients plus” for basic skin care in the European guideline [4]. 

With attention to the relevance of a loss-of-function *FLG* gene mutation or AD for the risk of developing sensitization to food allergens on the one hand [23, 24, 25] and to external ingredients on the other [26, 27], the guidelines on AD indicate that basic skin therapeutics should not contain allergenic ingredients such as food proteins and common contact allergens. The latter include, in particular, lanolin alcohols, cetylstearyl alcohol, and the preservative methylisothiazolinone, which, however, according to an amendment to the EU Cosmetics Regulation, may no longer be used in leave-on cosmetics since 2018 and has been limited to a concentration of 15 parts per million (ppm) in rinse-off products [28]. In contrast, the much-discussed parabens rarely trigger contact allergy as preservatives in cosmetics according to current data and can therefore even be recommended for cosmetics [28]. Fragrances, on the other hand, are among the frequent agents causing contact allergy. However, the group of the most frequently triggering substances is also subject to constant change here. Since atranol and chloroatranol as well as hydroxyisohexyl-3-cyclohexenecarboxaldehyde (HICC) have been banned as ingredients in cosmetics in EU countries in 2019 [29], isoeugenol has currently been primarily identified as a contact-allergy triggering fragrance by the “Informationsverbund Dermatologischer Kliniken” (IVDK) [29]. 

## Conclusion 

Genetic predisposition and inflammatory processes of the skin in AD influence the complex interaction of proteins such as filaggrin, their degradation products, and protein complexes such as tight junctions in the expression of the epidermal barrier. Consistent basic skin therapy to strengthen the skin barrier remains a fundamental part of the treatment regimen for AD, although its ingredients should be critically evaluated with respect to allergy risk. Furthermore, current RCTs do not provide evidence that the development of AD in the first years of life can be prevented by early use of basic skin therapy. 

## Funding 

None. 

## Conflict of interest 

L.M. Roesner has received institutional project funding and honoraria for scientific presentations from Novartis. 

A. Heratizadeh has received honoraria for scientific lectures and/or consulting from the companies Sanofi, Novartis, Leo, Meda, Hans Karrer, Nutricia, Pierre-Fabre, Abbvie, Lilly, and Beiersdorf and a travel grant by the company Janssen. 
